# Blockchain for Future Wireless Networks: A Decade Survey

**DOI:** 10.3390/s22114182

**Published:** 2022-05-31

**Authors:** Tejal Rathod, Nilesh Kumar Jadav, Mohammad Dahman Alshehri, Sudeep Tanwar, Ravi Sharma, Raluca-Andreea Felseghi, Maria Simona Raboaca

**Affiliations:** 1 Department of Computer Science and Engineering, Institute of Technology, Nirma University, Ahmedabad 382481, Gujarat, India; 20ftphde39@nirmauni.ac.in (T.R.); 21FTPHDE53@nirmauni.ac.in (N.K.J.); 2 Department of Computer Science, College of Computers and Information Technology, Taif University, P.O. Box 11099, Taif 21944, Saudi Arabia; alshehri@tu.edu.sa; 3 Centre for Inter-Disciplinary Research and Innovation, University of Petroleum and Energy Studies, P.O. Bidholi Via-Prem Nagar, Dehradun 248007, Uttarakhand, India; ravisharmacidri@gmail.com; 4 Faculty of Electrical Engineering and Computer Science, Stefan cel Mare University of Suceava, 720229 Suceava, Romania; 5 National Research and Development Institute for Cryogenic and Isotopic Technologies—ICSI Rm. Valcea, Uzinei Street, No. 4, Raureni, 240050 Rm. Valcea, Romania; simona.raboaca@icsi.ro

**Keywords:** wireless networks, security, privacy, blockchain, distributed ledger technology

## Abstract

The emerging need for high data rate, low latency, and high network capacity encourages wireless networks (WNs) to build intelligent and dynamic services, such as intelligent transportation systems, smart homes, smart cities, industrial automation, etc. However, the WN is impeded by several security threats, such as data manipulation, denial-of-service, injection, man-in-the-middle, session hijacking attacks, etc., that deteriorate the security performance of the aforementioned WN-based intelligent services. Toward this goal, various security solutions, such as cryptography, artificial intelligence (AI), access control, authentication, etc., are proposed by the scientific community around the world; however, they do not have full potential in tackling the aforementioned security issues. Therefore, it necessitates a technology, i.e., a blockchain, that offers decentralization, immutability, transparency, and security to protect the WN from security threats. Motivated by these facts, this paper presents a WNs survey in the context of security and privacy issues with blockchain-based solutions. First, we analyzed the existing research works and highlighted security requirements, security issues in a different generation of WN (4G, 5G, and 6G), and a comparative analysis of existing security solutions. Then, we showcased the influence of blockchain technology and prepared an exhaustive taxonomy for blockchain-enabled security solutions in WN. Further, we also proposed a blockchain and a 6G-based WN architecture to highlight the importance of blockchain technology in WN. Moreover, the proposed architecture is evaluated against different performance metrics, such as scalability, packet loss ratio, and latency. Finally, we discuss various open issues and research challenges for blockchain-based WNs solutions.

## 1. Introduction

**Figure 1:**
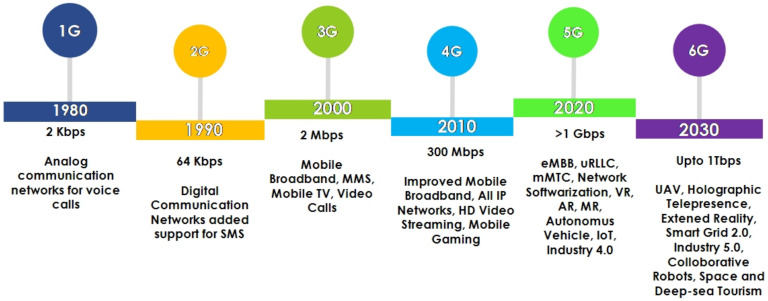
Evolution of wireless networks.

The landscape of wireless networks (WNs) is continuously expanding as a fast-growing technology with innovative features, such as flexibility, mobility, lack of wiring, etc. Their usage is increasing in diverse smart applications, such as smart cities, e-healthcare, intelligent traffic management, smart agriculture, autonomous vehicle, smart retail, and smart grid [1]. It is becoming an integrated part of people’s everyday life for day-to-day activities where a sender can transmit essential data to the receiver without using any physical medium (cables) [2]. Recent technological inclination in the WNs provides several benefits, such as ubiquitous high data rates, low latency, and high bandwidth, along with various limitations, such as security, privacy, reliability, authenticity, integrity, and scalability that can hinder the performance of WNs-based applications. To overcome the aforementioned issues, the scientific community has adopted effective radio resource management and modern technology, such as artificial intelligence, blockchain, quantum communication, etc., that offers better network performance in every next-generation wireless network. Figure 1 shows the evolution of wireless communication technologies that started in the late 1970s. It took almost 50 years for WNs to evolve from the 1st generation to the 5th generation to deliver a progressive quality of services (QoS) to an individual and the nation’s capital economy [3,4].

The first generation (1G) WN introduced in 1979 was meant to initiate voice communication between individuals using analog signals. Unfortunately, the success of 1G was quelled due to its several limitations, i.e., poor voice quality, high battery consumption, prone to security attacks, and limited capacity. Moreover, an adversary can perform clone and masquerade attacks and easily intercept the communications between two parties [5]. To mitigate the aforementioned problems in 1G, second generation (2G) introduced digital communication, such as a global system for mobile communication (GSM) and general packet radio services (GPRS). It offers features such as text and multimedia messages and data services with a transfer rate of 40 kbits/s. Additionally, it enhances the reliability of the 2G systems by providing error detection, and correction mechanisms [6]. However, with the internet services and multimedia platform, 2G does not facilitate satisfactory data transmission rates. Additionally, there are several security issues in 2G, such as illegal interception, message spamming, and false information injection. Hence, it is recommended by many technology makers and innovators to stop using 2G systems.

To overcome the limitations of the 2G system, the third generation partnership project (3GPP) has deployed third generation (3G) networks that came up with asymmetric and symmetric traffic, global roaming, and packet-circuit switching to enhance the performance of WNs. In addition, the 3G network offers technologies, such as enhanced data rates for global evolution (EDGE), code division multiple access (CDMA), and early development of long-term evolution (LTE) that offer high data rates (14 Mbps), which raises the connectivity of mobile devices and improves the existing cellular systems. Furthermore, with the availability of IP-based communication, many users worldwide are getting connected by 3G networks to use semantic web services. However, it also raises different security vulnerabilities, such as denial-of-service (DoS), overbilling, and signaling-level attacks [7]. To overcome these issues, the international telecommunication union (ITU) has fostered the development of the fourth generation (4G) network that makes efficient use of the radio spectrum and increases the capacity, data rates, and bandwidth to deliver low latency multimedia services [8].

Similar to other legacy systems (1G–3G), the 4G network is also leveraged by several security threats and vulnerabilities, such as manipulation of access points, distributed denial of service (DDoS), data integrity, and replay attacks that deteriorate the QoS of 4G-based applications. To tackle such security hindrances, the previous generation WNs (2G to 4G) offer several security solutions, such as configuring the first line of defense by installing firewalls and intrusion detection systems, secure data by encapsulating, encryption and authentication, and incorporating demilitarized zones to protect sensitive data and critical infrastructure from the adversaries [9,10]. However, there is an increase in privacy concerns as user demands are increasing. When a user uses wireless communication to connect to the Internet, it leaves many footprints that an adversary can collect from different WNs-based applications to perform user tracking and social engineering attacks. The development of the internet-of-things (IoT) technology enables portability and more openness to the wireless network. Since a portable device is easy to attack and track instead of an entire infrastructure of the organization, it increases the privacy leakage issues in various technology, such as Bluetooth, Wi-Fi-based laptops, and smartphones [11,12].

5G has added another dimension to the WN by satisfying the user’s demands of high data rates, reliability, scalability, and low latency communication [13]. The primary objective of a 5G network is to transform a standard cellular network into an intelligent network by incorporating AI, blockchain, edge computing, and IoT technologies. It also brings effective radio access techniques, such as massive multiple-input multiple-output (MIMO), device-to-device (D2D), millimeter-wave (mmWave), and ultra-densification connectivity, which prolongs the user scalability in WN [14,15]. However, the 5G network has abstracted design principles and is not appropriately documented; as a result, there is a high risk that malicious adversaries can maneuver the standards and regulations of a 5G network [5,16]. Additionally, integrating modern technologies with WN creates a different horizon of challenges, such as lower network resiliency, data integrity, downtime, single-point failure, coordinated attacks, and unauthenticated access control [17].

One of the plausible solutions to overcome a few of the above-mentioned security issues from WN is to adopt cryptographic techniques, where most of the WN-based applications and devices use end-to-end encryption by incorporating asymmetric and symmetric key encryption, message digest, and hashing [18]. However, to fully secure WN from attackers, we need a stronger and considerable size key length, which is computationally expensive and not feasible. Though with modern computing capabilities, one can generate such keys and secure the WNs. However, the problem lies in sharing the keys with communicating parties, which formally use a public channel, i.e., the internet, to share the keys. The attackers can manipulate those public channels, where they can access the private keys and intercept the ongoing communication between the sender and receiver [19]. This affects the security of the WNs and imperils the privacy of the end-users. Hence, there is a requirement for a robust technology, i.e., the blockchain, which can integrate with the WNs to relieve the security and privacy constraints [20,21].

Blockchain technology has an immutable decentralized ledger that can securely store the sensitive information of WN applications in such a way that it complicates the process of manipulation by the attackers [22]. Currently, it is embraced by various WN-enabled smart applications, such as financial, smart homes, smart grids, smart supply-chain management, and smart cities, to ensure secure communication while sharing the data between different participating entities of WN [23,24]. Further, the decentralized nature of blockchain makes the technology transparent and more reliable. This is because a member of the blockchain can see transactions made by the other blockchain member. Additionally, it is inclined toward concrete cryptographic public and private keys to secure each blockchain transaction. Therefore, it is resistant to various security and privacy issues, such as data injection and data tampering attacks, and overcomes the issue of single-point failure [25]. The integration of WN and blockchain has great potential, especially for mission-critical applications, such as e-healthcare, smart factories, public safety, and military services that require constant supervision against security threats. In addition, it also offers security to ensure interoperability and trust between complex sub-systems of smart applications.

To facilitate the integration of blockchain and WN, many researchers have proposed several state-of-the-art advances in blockchain-enabled WN. For example, Nguyen et al. [10] presented an extensive discussion on different opportunities that blockchain has brought to the world of 5G and future generation wireless networks. However, they have not discussed the critical shortcomings of blockchain in WN, such as security vulnerabilities and privacy concerns. Further, Wang et al. [26] introduced a comprehensive study of blockchain radio access network (B-RAN) based framework for 6G. They further elaborated on the necessity of a consensus mechanism, digital contracts, inter-network data sharing, and a trust model in WN to preserve the privacy of the authenticated users. Unfortunately, most of the integration between blockchain and WN specifies the partial aspects of security and privacy issues in WN. Many researchers have proposed blockchain-based solutions for secure wireless communication. However, very few of them discussed security issues and their countermeasures in depth. Thus, there is a requirement to follow a proactive way and consolidate emergent research works toward privacy and security issues of WNs. Hence, this paper highlights the security and privacy aspect and its effect on future WNs with possible solutions by resorting to blockchain technology.

### 1.1. Motivations and Novelty

The motivations of the proposed survey are as follows.

- Despite the essential benefits of WN, it is still imperiled by various security and privacy issues that impede its widespread applications in smart homes, smart cities, smart vehicular communication, and many more.
- Many researchers across the globe have provided their resistant solutions to confront the security and privacy issues of WN. However, the attackers are constantly upgrading their motives and formulating newer attacks that are challenging to tackle with the existing solutions.
- There is an imperative need for a technology, i.e., blockchain, which can overcome the security and privacy prospect of the WN by offering an immutable ledger and a decentralized and transparent network. However, the scientific community has not fully explored the integration of blockchain into the WN. Mostly the existing survey presents the integration of blockchain in a specific WN application and explores a few WN-based security attacks.
- This motivates us to write an exhaustive survey that investigates different security and privacy aspects of WN along with their blockchain-based solution. In addition, we proposed a taxonomical representation of different WN attacks and their possible solutions. Further, the proposed blockchain and a 6G-enabled WN architecture encourage to incorporate blockchain in every WN application.

### 1.2. Survey Contributions

Presently, there are various survey papers that exist in the literature, but as per our knowledge, very few of them entirely cover the security and privacy issues in WN. Therefore, the following are the contributions of this paper.

- This paper presents a systematic and comprehensive survey on WNs by exploring the security and privacy issues in various WN-enabled smart applications. It also bridges the gap between security and privacy issues by utilizing blockchain-based solutions.
- A taxonomy is proposed that contains a detailed description of security attacks and their countermeasures with the available security and privacy solutions of WNs.
- A blockchain-based WN architecture is proposed with layer-wise in-depth discussion.
- Finally, the paper highlights several open issues and research challenges for blockchain-based security and privacy solutions in WN.

### 1.3. Highlights of the Proposed Survey

The overall highlight of the proposed survey is as follows.

- Identifying the security and privacy issues with a different generation of WNs, i.e., 1G, 2G, 3G, 4G, and 5G, along with their existing solutions.
- A comprehensive explanation of different attacks in modern generation wireless networks, i.e., 4G, 5G, and 6G, discussed how they dampen the performance of the WN-based applications and their existing solutions proposed by the research community across the globe.
- Further, the influence of blockchain technology is explored to alleviate the security and privacy issues from WN-based applications.
- Then, an exhaustive comparative analysis of the existing survey with the proposed survey is formulated to showcase the importance of the proposed survey.
- The OSI model is a core on which the entire WN is operated; therefore, we explore active and passive attacks in each layer of the OSI along with their blockchain-based solutions. To support that, we propose an exhaustive taxonomy to provide an illustrative representation of each attack in the OSI model.
- Moreover, we present a blockchain and 6G-enabled WN architecture to confront the security and privacy issues of the WN. Then, the proposed architecture is evaluated with different performance metrics, such as packet loss ratio, scalability, and latency.
- Lastly, we mention open issues and research challenges in blockchain-based WNs to encourage other researchers to find novel solutions to mitigate the security and privacy challenges of the WNs.

### 1.4. Methods and Materials

This paper presents an in-depth systematic study on the security and privacy perspective of WNs using blockchain technology. First, we refer to distinguished digital libraries, such as Springer, Google Scholar, ACM, Science Direct (Elsevier), technical blogs, and IEEEXplore for research articles to gather high-quality research articles. Then, we explore different keywords, such as *"wireless networks, security and privacy in wireless networks, blockchain and wireless networks, security issues in wireless networks and blockchain, integration of blockchain and wireless networks"*, to collect qualitative literature up to 12 March 2022. After collecting various literature, analysis is performed on the available literature and we propose an exhaustive taxonomy on various security threats that impede wireless networks and their blockchain-based solutions. Then, a novel layer-wise WNs architecture is proposed using blockchain technology to tackle the security issues of WN.

### 1.5. Key Take-Aways

The key concepts that can be taken away by readers/researchers from the proposed survey are as follows.

- The reader gets an idea of detailed concepts of WNs and their evolution from a security and privacy perspective.
- This article presents how blockchain handles security and privacy issues in WNs for various smart applications, for instance, traffic monitoring, smart cities, smart home, smart grids, smart industries etc.
- Readers/researchers can get the gist of possible security attacks in WNs that resists blockchain.
- This paper delivers the open research issues and future directions for further enhancement in WNs while using blockchain technology.

### 1.6. Survey Organization

The rest of the paper is organized as follows: Section 2 highlights the background of WNs, blockchain technology, security in WNs, and influence of blockchain technology in WNs. Section 3 highlights the proposed taxonomy for security and privacy perspective in WNs. Then, Section 4 presents a layer-wise blockchain and a 6G-enabled WNs architecture. Section 5 discusses the result and analysis. Section 6 discusses the open issues and future research challenges. Finally, Section 7 concludes the paper.

**Figure 2:**
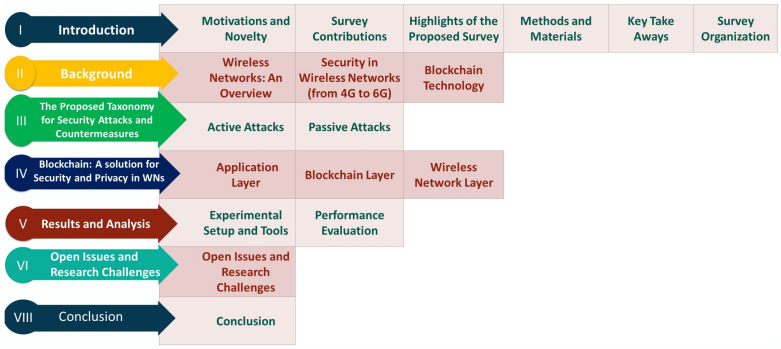
Organization of the paper.

Figure 2 shows the organization of the paper, which gives an overview of the entire article and allows the readers to read a particular section for their research. Abbreviations presents all acronyms used in the paper.

## 2. Background

This section highlights the background of WNs, layer-wise security issues in WNs, blockchain technology, and integration of blockchain with WNs.

### 2.1. Wireless Networks: An Overview

The WNs evolved in a short span of time, witnessing explosive growth in the sector of industry, healthcare, science, and technology by pervasively connecting them. Since the 1970s, newer generations of WN have been introduced, which adroitly improve people’s quality of life by providing productive services, such as voice calls, multimedia services, remote connections, on-demand, intelligent services, and many more. In 1979, the first cellular WN 1G was introduced, but it had low voice quality, higher interference, and no encryption mechanism was applied for secure communication. Then, with primary progressions, other generations (2G, 3G, 4G, and 5G) of cellular WNs were developed to add value to telecommunication and network service [3]. 2G provides a few imperative mobile call advancements, with encryption mechanisms, such as improving voice quality and reducing cross-talk [4]. On the contrary, 3G networks are faster and capable of transmitting data at a higher rate (maximum download speed 7 Mbps). They facilitated end-users to record video calls, watch TV online, surf the internet, and play online mobile games for the very first time [27]. Moreover, IoT-enabled devices become the center of social connectivity in 3G by using IP-based communication, but it also raises concern for security vulnerabilities [28].

4G has become the first generation to use long-term evolution (LTE) technology that improves the data rate and QoS of WNs. Moreover, 5G has replaced 4G with various changes, such as enhanced data rates (1 Gbps), low latency (100 ns), mobility range (100–500 km/h), etc., for better network coverage and reliability [29,30]. The communication latency in 5G has decreased substantially, resulting in fast download and upload speeds. Although 5G networks are becoming a reality, technologists have already started to be engaged with future WNs, i.e., 6G, which anticipates putting greater prominence on wearable technologies, unmanned aerial vehicles (UAVs), 3D networking, and wireless power transfer to amplify people’s quality of life [31]. However, the radio resources used by the WNs operators are entirely open to security attacks, and therefore there is a need to explore and examine such attacks to stop them before they jeopardize the WN systems.

### 2.2. Security in Wireless Networks (From 4G to 6G)

**Figure 3:**
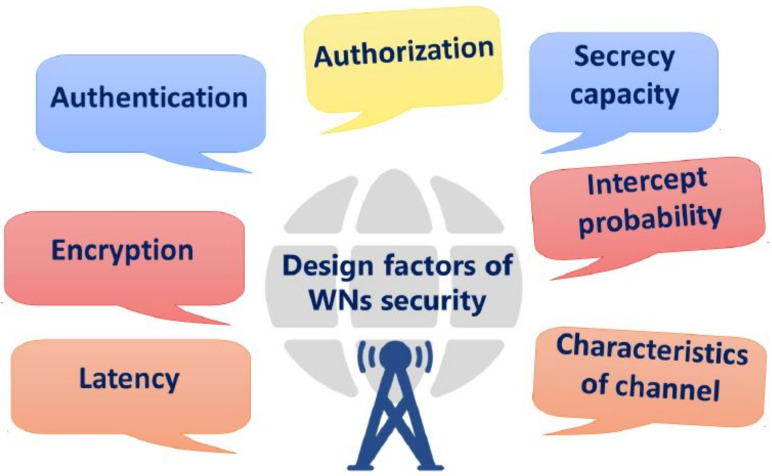
Design factors of wireless networks security.

This section highlights network security issues associated with different generations of WN, such as 4G, 5G, and 6G. In WN, a sender can share information via the Internet with the receiver. The Internet has an intricate design principle using network devices such as routers, switches, hubs, and cables, connected with simple topologies without a stronger security mechanism. This entices the attackers to scan the network devices and interfaces to find potential vulnerabilities which can further be exploited for their own benefit [32,33]. Thus, security and privacy play an essential role in protecting user data in the wireless medium [34]. To overcome that, security specialists have proposed several design factors that pave the way in thwarting the malicious attempts of the attackers. Figure 3 shows the design factors, such as authorization, authentication, encryption, intercept probability, and channel characteristics, that confront the security attacks and improve the reliability of the WN communication. A summarized explanation of each design factor is given as follows.

- Authentication—A standard example of WN is the internet, where tons of internet services serve the end users. A sender sends confidential information from these services to the receiver, which in return, the sender expects that the information reaches the correct receiver. Thus, before sending the data, both users have to authenticate themselves for reliable communication. Formally, authentication states that a user has to validate who he/she claims to be with the help of authentication factors, such as a strong password, personal identification number (PIN), one-time password (OTP), and biometrics. However, the attackers can attack the single layer authentication; for example, a password can be cracked using dictionary attacks, OTP can be brute-forced, and biometrics can be manipulated using masterprints or techniques, such as image processing, which generates similar finger prints of the authentic user. Therefore, multi-layer authentication systems are adopted by several organizations to secure their sensitive resources and provide seamless services to the users without any security hindrances.
- Authorization—Once the user in the public internet is authenticated, he/she can utilize various internet applications. However, from the security perspective, an attacker can impersonate the authentic user to maliciously read and write confidential information of the validated user or may use the services that are not meant for him. Therefore, there is a need to regulate access control mechanisms after authenticating the user, permitting only authorized users to access the system’s services and resources. For that, the administrator has to assign roles and permission to the legitimate user in the access control list. For example, a person can authenticate himself by inserting a username and password into the website; once authenticated, based on the roles and permission assigned, he can access or deny the further services in the website. This helps in poising the security and privacy of the WN-based application.
- Encryption—Authentication and authorization help in preserving the privacy of the system. For instance, a web application utilizes the WN to transmit messages from one user to another. One can guarantee that the users who are enrolled with the application are authenticated and authorized to use this service. This is because they are validated and verified by the authentication scheme and authorization mechanism prior to using this application. Nevertheless, the security is violated when the message is in transit; an adversary can access the transit message and try to manipulate it, disobeying the data integrity principles. Therefore, there is a need for incorporating proper encryption standards that obfuscate the message in a way that is not readable by the attackers. There are various encryption methods available, such as public and private cryptosystems comprising advanced encryption standards (AES), Rivest–Shamir–Adleman (RSA), blowfish, triple data encryption standard (DES), and many more. Further, to augment the user’s security and privacy, encryption algorithms can employ hashing algorithms, such as secure hash algorithm (SHA) and message digest, that strengths the WN security.
- Characteristic of channel—The aforementioned design factors are for higher-layer WN applications, but with the current exploration of radio frequencies, the attackers dwell in the physical layer security, wherein they exploit radio waves to intercept the ongoing communication. Thus, it is indispensable to understand the wireless channel and secure it by analyzing characteristics, such as bandwidth, data rates, channel quality indicators, i.e., signal-to-noise ratio (SNR), the reference signal received power and received signal strength indicators of the channel. Furthermore, a message generated at the sender machine has to pass through the dynamic wireless channel, which is time variant and has a lot of obstructions, such as interference, multipath propagation, delay, attenuation, path loss, and fading, which deteriorates the data rates of the WN. Therefore, an attacker tries to investigate such indicators to proliferate their physical attacks that dampen the performance of the wireless communication. Hence, it is essential to reinforce the wireless channel with effective channel coding, equalization techniques, and embracing physical layer security.
- Secrecy capacity—The notion of security and privacy in WN is not limited to studying the application and middle layer security, but also needs to investigate different malicious intent propagated at the physical layer. One such mechanism is the secrecy capacity, which is intrinsically associated with the channel capacity, where the channel is the broadcasting or transmitting the message from the legitimate user. Here the intended receiver is treated as an illegitimate user or an eavesdropper who is trying to intercept and decode the message from the legitimate user. An eavesdropper can decode the message if the channel gain between the transmitter and an eavesdropper is higher than the channel gain between the transmitter and receiver. This also means that the eavesdropper has a higher channel capacity resulting in decoding the messages of the users in close proximity. Therefore, as a network analyst, it is imperative to analyze channel gains and data rates to eliminate the eavesdropper from future communication.

Despite the aforementioned design factors of WN security that are precisely implied to secure the WN, attackers can still prolong their malicious intent to jeopardize the performance of different interfaces of WN, i.e., 4G, 5G, and 6G. Therefore, this subsection presents the security issues and their relevant solutions that are proposed by the scientific community globally.

#### 2.2.1. Security in 4G

To mitigate the issues of a 3G network, the 4G network has been launched, which is a packet-switched network with a network capacity of at least ten times 3G network. It provides higher bandwidth, up to 100 Mbps, and service personalization to meet the demands of diverse users to provide highly customized services. The 4G network has high usability and global coverage that fulfills the users anytime and anywhere [35]. Nevertheless, the 4G technologies appear much more promising for QoS and user experience; some shortcomings still need to be addressed, such as network security and privacy. Worldwide interoperability for microwave access (WiMAX) is a well-known standard for 4G-based wireless broadband communication (IEEE 802.16). It uses orthogonal frequency division multiplexing (OFDM) and the space–time block coding (STBC) model with cryptographic encryption to secure over-the-air communication [36]. However, despite having a secure and robust WiMAX architecture, it still does not ensure end-to-end security against numerous attacks such as key exchange and management, DoS, scrambling, and masquerading attacks. Similar to WiMAX, long-term evolution (LTE) is another standard that enables user equipment (UE), such as laptops and smartphones, to connect to the base 4G-based base station via an evolved-universal terrestrial radio access network (E-UTRAN). The security architecture of a 4G LTE system is continually evolving and improving by incorporating secure signaling between user devices and the BS, strong authentication, and key agreement protocols [37]. However, restraining attacks at different levels of open system interconnection (OSI) is challenging and has always been a matter of concern in the 4G system. Moreover, maintaining high-level security by monitoring, analyzing, and troubleshooting makes the recurring cost more expensive. This is because more and more people are adopting smart devices for various services, such as commercial banking, mobile web access, IP telephony, video conferencing, and many more in highly insecure environments, such as wireless hotspots and public Wi-Fi (such as cafes), etc. Deploying the above-mentioned security schemes over such places is deceptive, as the 4G network is an IP-based network that is open to several vulnerabilities that invite security risks and threats associated with different 4G-based services [38].

To overcome the aforementioned security threats, the scientific community has proposed different security solutions. For example, Wu et al. [39] discussed the structure and advantages of the 4G network in wireless communication. They identify WN security problems and present secure communication solutions for 4G. Moreover, Ref. [40] analyzed security and privacy issues in 4G-LTE and IoT networks. For that, they explored a DoS attack using open-source identity-defined radio (SDR). After analyzing security issues, they proposed security solutions for future WNs. Jasim et al. [41] analyzed various encryption algorithms, such as ZUC, SNOW 3G (a stream cipher algorithm), and advanced encryption standard (AES), that provide robust security in 4G systems. In addition, they presented cryptanalysis techniques that improve data security. However, there is a need for an upgraded technology that not only provides seamless data rates, but also protects from modern vulnerabilities and security threats. Therefore, a 5G-based wireless communication is proposed, which is discussed in the following topic.

#### 2.2.2. Security in 5G

The 5G wireless network is the next-generation mobile communication system initialized first by NASA in April 2008. After ample R&D conducted by numerous countries, it was commercially launched in December 2019 by the American telephone and telegraph company (AT&T) [42]. Nowadays, 5G has become truly ubiquitous in the field of mobile communication systems. It unlocks features such as service optimization, effective decision-making, and better end-user experience [34]. 5G speeds range from 50 Mbps to up to 1 Gbps with the spectrum of sub 6 GHz (5G macro optimized). In addition, it can achieve the speed of 4 Gbit/s in MIMO with the mmWave bands and carrier aggregation. Network latency would be around 1 to 2 ms, which is 50 times lower than 4G.

The previous generation (2G, 3G, and 4G) of WN are compatible with each other, which means 3G is evolved by incorporating the technologies of 2G; similarly, 4G is evolved using 3G technologies. However, the 5G technology has adopted new protocols and standards to leverage promising use cases, such as enhanced mobile broadband (eMBB), URLLC, and massive machine-type communications (mMTC). However, the 5G network uses conventional security protocols, such as secure hypertext transfer protocol (HTTPS) and secure socket layer (SSL); this raises anxiety, as attackers are well versed in internet technologies. Therefore, they can search for different vulnerabilities residing in the security protocols and the applications used by the 5G end users from the databases, such as common vulnerabilities and exposures (CVE). For example, most of the web application uses JavaScript object notation (JSON) to represent the structured data; the attackers can maneuver the JSON object to proliferate the DoS attack and gain control over the network devices. Furthermore, the technologies used in the 5G network are harder to manage; hence, there is a wide possibility that the services are misconfigured. Consequently, the attackers can target the incorrect configuration of such services or equipment [43].

To overcome the aforementioned security issues, Park et al. [44] discussed various technological aspects and services for 5G security, such as authentication, integrity, availability, confidentiality, and non-repudiation. Qian et al. in [45] studied the security aspects of 5G WN. They discussed the potential attacks and the security service requirements needed for the new uses cases of 5G WN. Their prime focus is to blend the new security features in the different technologies of 5G WN, such as heterogeneous networks, software-defined networks, IoT, and MIMO. Further, it is necessary to verify the routing information between various nodes of the WN. The attackers can easily intercept the data traffic and manipulate the WN application if the route is not secure. Therefore, adopting a secure route optimization protocol by formulating secure authentication, key exchange, and privacy protection can significantly improve WN security.

#### 2.2.3. Security in 6G

The main objective of 5G WN is to connect different smart devices to offer automation in smart cities, smart industries, and smart vehicles systems. The next-generation WN, i.e., the 6G network, presents a fundamental shift by unifying digital technologies and the human world to provide intelligent systems that redefine human lives. Evidently, the expansion of the 6G network is based on the technologies of the 5G network, such as AI, machine-to-machine (M2M) communication, digital twins, and many more. This enables the 6G developers to analyze the physical world closely by simulating it and take practical actions back to the physical world. It offers high data rates of up to 1 Tbps, high reliability (99.99999%), high scalability, low latency (1 ms) using further enhanced mobile broadband (FeMBB), extremely reliable and low-latency communication (ERLLC/eURLLC), and enhanced ultra-reliable low latency communication between different service providers and end-users. The low power consumption feature of 6G makes it 10 times more energy efficient compared to 5G. However, the security concern of the 6G network is still a matter of discussion, where malicious adversaries can impede the tight coupling between a 6G network and modern technologies. Some of the security threats in 6G are the privacy of users and data, artificial intelligence (AI) attacks, and trust violations [3].

Since the 6G network is still not developed and publicized, very minimal literature is available that specifies the security and privacy of the 6G network. Here, we incorporated some of the contribution that researchers have presented to tackle the security issues in a 6G network. For instance, Wnag [46] identified key areas of 6G, such as intelligent radio, 3D intercoms, distributed AI, and real-time intelligent edge. Moreover, they presented a survey on security and privacy issues in 6G networks. Porambage [3] discussed 6G security challenges with various 6G applications, such as trust management problem in smart grid, scalability and automation issue industry 5.0, fake experiences in extended reality, privacy protection in holographic telepresence, and unarmed aerial vehicle (UAV)-based mobility. Furthermore, they explored distributed ledger technology, quantum computing, and scalable AI/machine learning (ML) technologies with 6G and identified how it affects the security and privacy of 6G [47]. Despite the fact that the 6G network has indispensable capabilities, the security problem continues to hinder the end-user experience. The reason behind that is that the 6G network integrates many modern technologies such as AI, IoT, optimization, data analytics, and many more discuss the intelligent use cases of 6G, but an essential part of security is primarily missing from their fundamentals. Therefore, there is a strong requirement for a technology, i.e., blockchain that blends with 6G networks to provide a proactive mechanism to secure 6G WN against numerous security threats.

### 2.3. Blockchain Technology

**Figure 4:**
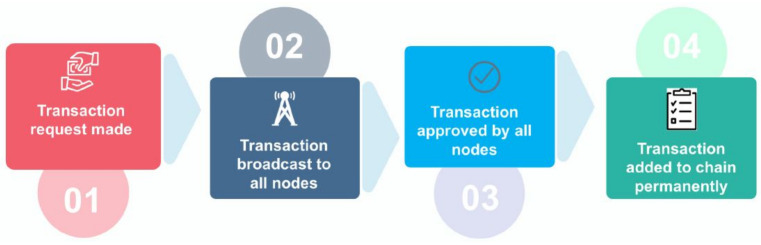
A workflow of the blockchain process.

Blockchain is a peer-to-peer (P2P) architecture that weakens the dominance of third-party intermediaries by utilizing decentralization with essential features, such as immutability, reliability, transparency, and security. The blockchain blocks are connected with each other to form a distributed ledger, where each block stores/maintains the hash of the previous block. Any minuscule change in one block reflects the difference in the hash of the other blocks. Therefore, blockchain technology is transparent and reliable against data integrity attacks. Moreover, the distributed ledger is secured by cryptographic techniques, such as digital signatures, hash, and public–private key pairs that validate each transaction whenever a new transaction is added to the blockchain [48,49]. Figure 4 shows a workflow of a blockchain transaction; wherein a transaction request is broadcast to all the nodes of the blockchain. In addition, digital signatures are used for user identity (a node can sign the document and broadcast it to all other nodes). Then, the private and public keys are used to verify the signature. Then, each block records this transaction to validate it by verifying the hash of the blocks. Moreover, all users connected to the blockchain contain the same updated copy of data which shows transparency within the network. After the successful verification, it is permanently added to the chain of blocks.

**Figure 5:**
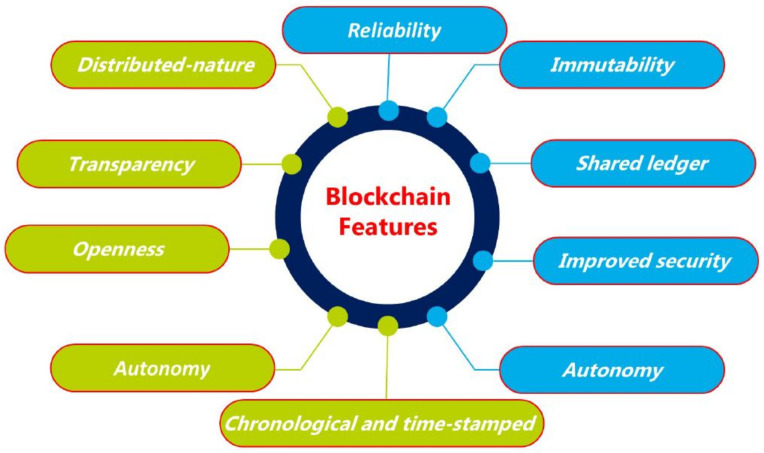
Blockchain features.

The distributed nature of the blockchain benefits the WNs in various ways, such as handling the single-point failure issues, incorporating trust mechanisms, secure access control, and preserving the user privacy [50]. It enforces the new security advances to protect the WN from modern security threats, such as cryptojacking and ransomware. Toward this goal, when the WN user publishes (store) the data on the blockchain, it is difficult for an adversary to modify them because of the immutability feature of the blockchain technology. In addition, blockchain immutability can find internal and external attackers by analyzing the change in the hash of the blocks [51,52]. Figure 5 illustrates unique features of blockchain that strengthen WNs.

#### 2.3.1. Blockchain Hierarchy

**Figure 6:**
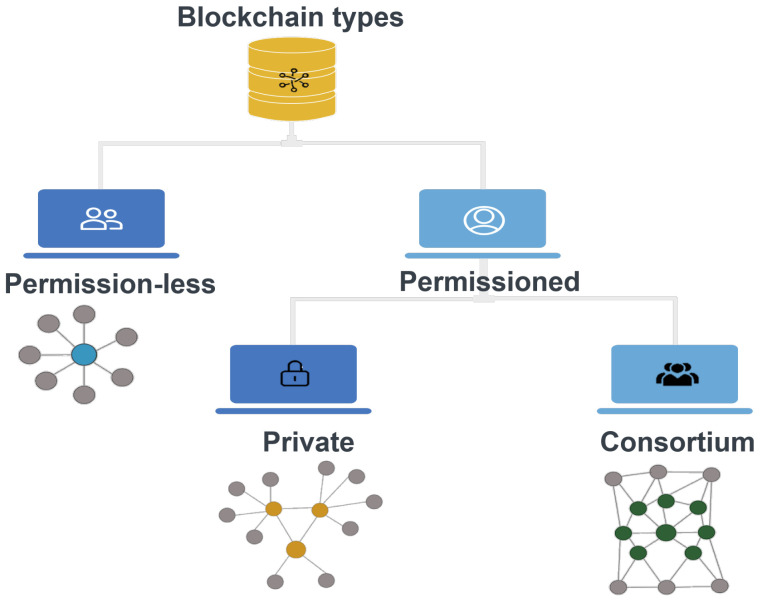
Hierarchy of blockchain.

As per the available literature, blockchain can be categorized into two broad categories: (i) permission and (ii) permissionless (i.e., public) [53]. Then, the permission blockchain is further classified into sub-type, private and consortium blockchain. In the permission blockchain (private blockchain), a user has to take permission from a trusted blockchain authority to participate in the blockchain network. Contrary, in the permissionless blockchain (public blockchain), any user can be a part of this network and continues to upkeep. A public blockchain is comparably more secure than a private blockchain; this is because a public blockchain is transparent, i.e., every blockchain member has an identical copy of the distributed ledger. Therefore, if some attacker tries to manipulate the ledger, it will be notified to each blockchain member, and consequently, we can find the malicious intender. There are various smart applications where public blockchain is being used, and one of the prominent examples is Bitcoin (i.e., the first cryptocurrency). Furthermore, the transaction in the public blockchain is secure, tamper-proof, and auditable [54]. Figure 6 illustrates the hierarchy of blockchain.

#### 2.3.2. Integration of Blockchain and WNs

Undoubtedly, the next generation WN has a blend of modern technologies that use virtualization and cloudification to handle an enormous amount of data generated by the WN users. Furthermore, the popularization of cellular networks, which use WN as their core component in driving cellular services, has seen a significant growth in data traffic. These voluminous data are unable to be handled by a single technology, and hence, the researchers have started to integrate multiple technologies, such as AI, IoT, automation, and many more, in WN to handle the WN services seamlessly. Since the inception of WN, the data have been the foremost important entity that served millions of users by connecting them around the world, offering on-demand services, entertaining with multimedia services, and many more. However, it has been observed that many a time, the en-route data of WN are manipulated by the attackers, increasing the unexpected behavior of the system. Therefore, there is a need for a concrete technology that has secure storage capabilities, where attackers cannot tamper with the data. Blockchain is one such promising technology with cryptographic techniques and a distributed ledger that is shared among the authenticated members of the blockchain that ensures safety and privacy for WNs [10]. Additionally, it facilitates a smart contract, a digitally secure signed program to establish a contract between two entities without the need for a third party for transaction settlement.

Multiple researchers are working on integrating blockchain technology with the WNs. For instance, Xu et al. [55] proposed a resource allocation scheme using blockchain underlying a 6G network. Due to the scarcity of spectrum in a 6G network, it is infeasible to efficiently allocate resources to different 6G WN users. Therefore, for efficient sharing and managing the resources, blockchain technology has been proposed that improves the performance of the 6G network, reduces administrative cost, and maximizes spectrum efficiency. Then, Verma et al. [56] integrated blockchain with 6G network using a smart contract. They used consensus protocol with a 6G interface that benefits service level agreement, trusted database, and fair access. Further, Wang et al. [57] analyzed the application of blockchain on the internet of vehicles (IoVs). In that, blockchain offers a decentralized and secure environment that resolves the issue of centralized problems and improves IoVs architecture.

From the perspective of security, researchers across the globe found different methods to overcome the security and privacy issues of WNs. For example, Zou et al. [32] analyzed security vulnerabilities and threats in wireless communications. They discussed various security attacks, protocols, and algorithms in WNs and studied efficient defense mechanisms for security improvements. They focused on physical-layer security and introduced a family of jamming attacks with their countermeasures. Boudguiga et al. [58] discussed security issues, such as confidentiality, integrity and availability in IoT. They studied blockchain-based architecture for IoT applications, such as smart homes, smart grids, and Industry 4.0. Then, Ferrag et al. [59] discussed existing authentication and privacy-preserving schemes for 4G and 5G networks along with taxonomy. They discovered research gaps in existing security mechanisms, such as attacks against authentication schemes, availability, and integrity. The paper presents a comparative analysis of existing security mechanisms and their countermeasures that resolve security and privacy issues in WNs.

With the upcoming standards and technologies of WN, the WN use cases are becoming more competent and intelligent, such as smart supply chain, smart healthcare, smart vehicles, and smart cities. However, incorporating intelligence in such applications can sometimes lure the attackers into targeting them. The attackers can employ an adversarial AI model to degrade the performance of the WN application. This is relatively an easy task by the attacker because there is no mechanism by which one can verify at the deployment time whether the AI training model is trustworthy or not. Hence, incorporating the blockchain in smart applications of WN can help in tracking every small change that has been made in the AI model. To mitigate the aforementioned issues, Pohrmen et al. [60] presented a survey on blockchain-based security aspects in heterogeneous IoT networks. They discussed basic blockchain concepts and how it is used to resolve the SDN-based IoT platforms. Further, Casino et al. [61] reviewed the blockchain-based applications through multiple domains, such as supply chain, healthcare, privacy, business, IoT, and data management.

Rouhani et al. [62] presented security methods, performance enhancement approaches, and decentralized applications based on smart contracts. They performed an exhaustive state-of-the-art comparison where they compared the blockchain-based outcomes with other existing solutions. Aggarwal et al. [63] surveyed the application of blockchain technology for smart communities. Then, Liu et al. [64] reviewed blockchain-based identity management systems. Wang et al. [65] examined a detailed study on blockchain-based IoT applications. They addressed the critical challenges of IoT and data security in WNs. Further, the technologies were compared in terms of applicability to the IoT scenarios. Then, Xie et al. [66] presented survey on blockchain for cloud exchange (cloudEXs). The conventional cloud exchange has to suffer from data manipulation attacks by third-party auctioneers; therefore, blockchain integration with cloudEXs was proposed. They presented the survey of blockchain technology with cloud exchange in terms of security and privacy. Wazid et al. [67] presented a survey on system models for a 5G-enabled IoT communication environment. They conferred security requirements and security attacks in WNs. After the analysis, they compared the existing security protocols in WNs. Next, Hewa et al. [68] explored the blockchain-based smart contracts applications and highlighted the future potential. They discussed challenges in smart contracts that need to be resolved before deploying them to large-scale applications.

Saraswat et al. [69] analyzed the blockchain-enabled federated learning (FL) approach for trust management among UAV swarms and ground stations. They proposed beyond 5G-based UAVs architecture and considered a case study on the internet-of-military-things (IoMT) ecosystem. Abualsauod et al. [70] analyzed UAV security using blockchain with ML techniques. They introduced a hybrid blockchain model for the reliability and security perspective. Grover et al. [71] proposed a comprehensive survey on blockchain-enabled VANET security solutions. They analyzed various security, privacy, application, resource management, reliability, and integration elements. They gave direction for blockchain-based security protocol in the VANET environment based on these. Pattewar et al. [72] discussed blockchain-based solutions for security and data privacy in the IoT system. They considered consensus algorithm-based techniques for different use cases, such as smart homes and smart cities. Wang et al. [73] discussed the security and privacy issue with blockchain-based solutions in the metaverse. They analyzed the security vulnerability based on the identity, network, data, and privacy. They also discussed the technologies, such as fuzzy vault, digital twin, game theory, and blockchain, to overcome the above-mentioned security issues.

Many researchers have presented a survey of blockchain-based solutions for security and privacy in WN. They have discussed the implication of blockchain in the existing wireless system with the use case and analyzed their solution. However, they have not discussed security vulnerability based on the different layers. In this paper, we analyze the existing blockchain-based solutions to mitigate the security attack in each layer of the OSI. After studying the current security and privacy issue, we propose an architecture enabling blockchain technology for WNs.

Table 1 represents a comparative analysis of the existing surveys proposed with blockchain-based WNs on various parameters, such as contributions and observations.

## 3. The Proposed Taxonomy for Security Attacks and Countermeasures

**Figure 7:**
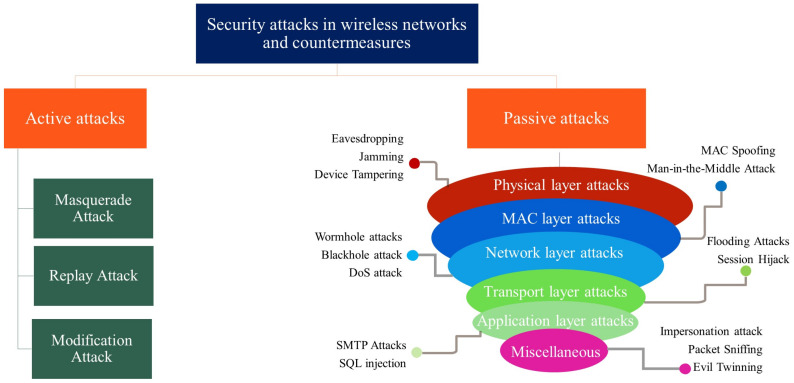
The proposed taxonomy for blockchain-enabled wireless networks.

Since the beginning, wireless communication systems have been susceptible to security vulnerabilities in every generation. Therefore, this section highlights the various security attacks on WNs and their countermeasures [75]. Figure 7 shows the proposed taxonomy for blockchain-based WNs from a security and privacy perspective. The section comprises various security attacks, such as wormhole attacks, session hijack, jamming, etc., and is classified into two main categories, i.e., active and passive attacks as per the available literature. Then, the countermeasures for each attack are discussed in detail from the perspective of WNs. In addition, Table 2 presents a detailed analysis of different countermeasures that are considered by researchers to handle security attacks in different layers of WNs. A detailed explanation of each attack is described as follows.

### 3.1. Active Attacks

In an active attack, the attacker modifies the message that sends from the sender to receiver. Malicious users can alter authorized data, such as source and destination address, timestamp, and user data. In addition, they can perform unauthorized transmission, modify the data to maneuver the behavior of the WN application, and block the critical resources (critical data, web server, and jamming the radio frequency) using DDoS attacks [76]; the following are examples of active attacks in WNs.

- Masquerade attack—A malicious user behaves like an authorized person to deprive the resources of the system [77]. They can capture authentication sequences and legitimately retrieve the access privileges to the target system. This attack creates a fake network identity that affects the financial transaction systems by phishing emails [78]. Furthermore, it makes counterfeit servers and sends data-stealing malware for their own malicious usage. In [79], the author proposed a signal strength fluctuation model to perform significant testing of masquerade attacks in a mobile ad hoc network (MANET). Moreover, using a masquerade attack, a malicious user can hack and remotely control modern vehicles. For that, ref. [80] proposed an authentication protocol that protects the electronic control units. Furthermore, Li et al. [81] introduced a blockchain-based solution using renewable hash chains that resist masquerade attacks in the identity authentication approach.
- Replay attack—In this attack, an intruder intercepts the communication and captures user data, then modifies it and sends it back to the destination to misdirect him [82]. It creates duplicate transactions in-network and gains information. Secure socket layer (SSL) or transport layer security (TLS) protocols, one-time passwords, timestamp methods, and session key encryption methods are used to prevent replay attacks [83]. Multiple researchers offer various solutions to prevent replay attacks for applications, such as vehicular ad hoc networks (VANETs), mobile ad hoc networks (MANETs), and IoT networks. For that, the author of [84] analyzed the existing schemes that prevent replay attacks in VANETs. In [85], the author proposed a model that overcomes replay attack breaches in authentication conditions of a smart city. In identity authentication scheme [81], Li et al. proposed a timeout mechanism that prevents replay attacks. Apart from these, Yavari et al. [86] introduced shared session values and freshly random values that create different session messages, which can be used to mitigate the replay attack probability.
- Modification attack—An attacker modifies some portion of the message transmitted from sender to receiver, which results in delivering corrupted and delayed messages [82]. It affects the data integrity and re-configuring of system hardware. A malicious user performs three types of modifications, i.e., change information, insert information, and remove existing information. To prevent this attack, encryption algorithms, traffic padding, and various messaging techniques, such as authentication code, sequence numbers, and checksums, are used [87]. Many researchers work to mitigate the issue of modification attacks in WN security. For that, [88] discussed how to overcome price modification attacks in smart grid applications. In [89], Al-shareeda et al. discussed a existing method that suppresses the modification of attack in VANETs. Moreover, Lin et al. [90] proposed a blockchain-based message authentication code and group signature mechanism that eliminates the effect of modification attacks for smart homes systems.

**Table 2:** Countermeasures for passive security attacks in different layers of WNs.

Layer	Attacks	Countermeasures	Ref
Physical layer	Eavesdropping attack	Limiting resources by network segmentation Avoid untrusted links Blocking unintended radiation in a computer using shielding	[91,92,93]
	Jamming	Anti-jamming systems Cryptographic Steganography	[94,95,96]
	Device tampering	Firewalls Encryption technique-Hash based message authentication code (HMAC) Access restriction File integrity monitoring	[97,98]
MAC layer	MAC Spoofing	Encrypted protocols Alert-enabled traffic monitoring Reverse address resolution protocol	[99,100,101]
	MiTM attack	Public-key authentication Strong WEP/WAP encryption on AP Use of a virtual private network (VPN) and Wi-Fi networks	[102,103,104,105]
Network layer	Wormhole attack	Packet leashes Separate route algorithm	[106,107,108]
	Blackhole attack	Redundant route method Time-based baited approach	[109,110,111]
	DoS attacks	Limiting broadcasting Protecting endpoints Rejecting fake traffic using firewalls and routers	[112,113,114]
Transport layer	Flooding attacks	Configuring the firewall Installing an intrusion prevention system (IPS) that identify network traffic patterns	[115,116]
	Session Hijack	Encrypt session id User authentications Secure cookies Ciphering the packets IPSec, and SSL approaches	[117,118,119]
Application layer	SMTP attack	SSL and TLS Inserting a security layer into the SMTP server prevent this attack	[120,121]
	SQL injection	Make more than one database user account Web applications firewall Limit privileges Adding input validation prevents SQL injection	[122,123]

### 3.2. Passive Attacks

In passive attacks, malicious users intercept and eavesdrop on the wireless communication channel to obtain unauthorized access. There are various passive attacks in WNs, which are discussed layer-wise as follows.

1. Physical layer attacks—This layer consists of data transmission, signal detection, encoding, modulation, and frequency selection tasks in the wireless medium [124,125]. A detailed description of each passive attack on the physical layer is given as follows.
  - Eavesdropping attack—It is also referred to as wireless sniffing, where an attacker eavesdrops over ongoing communications between sender and receiver [126]. This attack uses an eavesdropping device that monitors network activity, a listening port that records phone conversations, and a tapping transmission link. It silently impacts the performance of the WN in terms of privacy loss and identity theft [127]. Various methods are available to shove the eavesdropper from the authentic communication line, such as limiting the resources of the eavesdropper by network segmentation, avoiding opening untrusted links, and applying jammers or beamforming techniques to minimize the signal power of an eavesdropper. For that, many researchers provide various solutions; for example, [128] discussed eavesdropper attacks on 802.11ad mmWave systems. Moreover, ref. [91] prevented eavesdropping on radio-frequency (RF) and free-space optical (FSO) systems. In [92], Shi et al. proposed blockchain-based solution for dynamic honeypot system. They introduced the encryption algorithm RSA 2048-bit that resists eavesdropping attacks in the communication channel. In [93], Cheikhrouhou et al. came up with a blockchain-enabled secure localization algorithm that prevents eavesdropping attacks in IoT applications.
  - Jamming—It is a similar attack to the DoS attack, where the adversarial WN nodes obstruct legitimate communication by raising the interference or noise of the signal. It uses severe radio interference that makes the wireless channel busy, interrupts the ongoing communications, and reduces the signal-to-noise ratio (SINR), resulting in loss of communication [129]. It can be prevented with the help of an effective radio resource allocation mechanism [130], anti-jamming systems, cryptographic, and steganography techniques [131]. The authors of [94] overcame the jamming attack in the WNs control system by estimating the channel state estimation of the wireless channel. In [95], the author discussed anti-jamming methods for jamming attacks in VANETs, cognitive radio networks (CRNs), ZigBee networks, and GPS systems. Moreover, in [96] Danish et al. proposed a blockchain-enabled lightweight two-factor authentication mechanism for long-range wide area network (WAN).
  - Device tampering—It is the most straightforward way to attack the physical layer by making a modification in a WN device and extracting sensitive data from it [124]. The most popular way to perform this attack is to mirror a port of a medium access control (MAC) layer switch or hub. Once port mirroring is implemented, the data directed to the legitimate user are redirected to the malicious user. Such attacks can be controlled using firewalls, encryption mechanisms, hash-based message authentication code (HMAC), access restriction, and file integrity monitoring [98]. Moreover, Lee et al. [97] proposed blockchain-enabled data tamper-proof gateway architecture for secure resource management in smart home applications.
2. MAC layer attacks—The transmission of data packets is controlled by the MAC layer, using remotely shared channels [132]. The possible passive attacks on MAC layer are as follows.
  - MAC spoofing—It is a common attack in the MAC layer, where the attacker changes the MAC address of transmitted frames to spoof the security devices and protocols [133]. In this attack, malicious users spread malware, steal user information, and bypass the network access control systems. Encrypted protocols, alert-enabled traffic monitoring, and reverse address resolution protocol prevent MAC spoofing attacks [134]. In [99], the authors presented a comprehensive survey to detect and prevent MAC attacks on monitoring systems. Moreover, ref. [100] proposed a cooperative spoofing attack detection technique to identify MAC attacks. In [101], Islam et al. presented a blockchain-based secure data handover approach in non-orthogonal multiple access (NOMA) transmission schemes. They discussed a two-phase encryption algorithm to prevent MAC spoofing in WNs.
  - Man-in-the-middle attack (MiTM)—It permits an intruder to eavesdrop and preclude the communication between sender and receiver. Here, attackers modify the information, intercept and replace data between sender and receiver [135,136]. They steal user information such as card details and credentials of an e-commerce site, businesses, and financial applications. This attack makes interception by exploiting address resolution protocol (ARP) and spoofing the MAC addresses to mislead the data frame from their original path [137]. To mitigate the effect of the MiTM attack, various techniques are employed, such as public-key authentication, a WAP encryption mechanism, and the use of a virtual private network (VPN) [138]. The authors of [102] proposed a SQL injection, brute force attack, and cross-site scripting to avoid MiTM attacks. Further, in [103] the authors analyzed the MiTM attack in Wi-Fi networks, where they proposed a secure mechanism to prevent the content addressable memory (CAM) table of the wireless switch from the attackers. Further, Kulkarni et al. [105] discussed how to accomplished MITM attack in HTTPS. They proposed Nebulas blockchain technology-based solution to prevent MITM attacks in internet communication. In [104], Li et al. proposed a blockchain-enabled lightweight digital signature approach to secure UAV communication.
3. Network layer attacks—This layer is most vulnerable to various types of network attacks, which are discussed as follows.
  - Wormhole attacks—In this attack, the attacker creates a forged link between sender and receiver to drop or modify the data packets. The wormhole hole attack impacts confidentiality, availability, and data integrity [139] in applications such as VANETs, IoT networks, and MANETs. The packet leashes approach is used to prevent and defend against the wormhole attack. Moreover, separate route algorithms, watchdog models, and Delphi techniques are feasible solutions to avoid wormhole attacks [140]. In [106], the authors considered wormhole attacks as traffic attacks and presented trust-enabled techniques that detect and prevent such attacks in MANETs. The authors of [107] analyzed and resolved wormhole attacks in IoT networks by simulating the attacks in an IoT environment using NS2 simulation. In addition, Mohanty et al. [108] discussed security and privacy issues in payment channel network (PCN). They proposed Neo hashed time-lock commitment (n-HTLC) and a key encryption-based protocol that prevents wormhole attacks in the PCN.
  - Blackhole attack—In this attack, the router destroys and deletes all the messages that are supposed to forward to the legitimate destination (maybe the next router or intended receiver) [141]. Using this attack, the malicious user isolates the network and drops the incoming packets. To overcome the aforementioned issues, the authors of [142] used a time-based baited approach, and [143] proposed fake route request (RREQ) packets that find blackhole attacks in MANETs. Then, the authors of [109] analyzed various detection methods of blackhole attack [110] and proposed a hidden Markov model approach that prevents the attack in wireless sensor networks. Furthermore, Kudva et al. [111] presented a blockchain-based two-level detection system in the VANET routing protocol. This method eliminates blackhole attacks and calculates a trust score that increases VANET performance.
  - DoS attacks—The attacker often floods the data packets to the targeted access point, which shuts down the network and creates a resource starvation attack (DoS) [144]. In a DoS attack, the attacker sends spoofed, ping, and malformed packets to the target network, which slows down the network performance losing connectivity to the device. Using this attack, malicious users target sensitive data of the government, personnel, and the financial department [145]. Limiting broadcasting, protecting endpoints, and rejecting fake traffic using firewalls and routers prevent DoS attacks [146]. The bandwidth prediction technique [147], a simple and light-weight statistical detection and mitigation approach, can also be used to avoid DoS in WN applications [112,148]. Moreover, ref. [112] prevented flooding-based DoS attack in MANETs. Spathoulas et al. [113] proposed the use of lightweight agents in various IoT applications such as smart homes, smart grids, smart industries, etc. that detect DoS attacks. They offer blockchain-based smart contracts that resist DoS attacks in IoT botnets and ensure data integrity. As well, Hewa et al. [114] presented a security service blockchain (SSB) solution that mitigates DoS attacks in network slice broker.
4. Transport layer attacks—An end-to-end connection set-up is done for transferring the data packets, congestion, and flow control in the transport layer [149]. It uses transmission control protocol (TCP) and user datagram protocol (UDP). TCP is used for reliable data transmission while UDP reduces the overhead and latency. Due to their security vulnerability, these protocols suffer from flooding and session hijack attacks.
  - Flooding attacks—TCP sends numerous ping requests in this type of attack, whereas UDP sends several UDP packets. It creates network congestion to legitimate traffic and makes the system unresponsive [150]. Malicious users perform this attack using route requests (RREQ) or data flooding. A flooding attack is prevented by configuring the firewall and installing an intrusion prevention system (IPS) to identify network traffic patterns [151]. The authors of [152,153] presented TCP SYN flooding (half-open attack) prevention and mitigation techniques in a WN-based software-defined network. Moreover, ref. [115] proposed an OpenFlow-based TCP SYN authentication approach that overcomes the issue of flooding. Zhu et al. [116] proposed a decentralized blockchain solution that provides security in named data networking (NDN) of things by mitigating flooding attacks and data phishing.
  - Session hijack—In this attack, a session is taken away by an attacker between sender and receiver. In addition, the attacker takes off the victim’s session and behave like a legitimate user over the communication [154]. Session sniffing, cross-site scripting, and predictable sessions token ID are the most responsible parameters that perform session attacks. The adverse effect of this attack deteriorates the security of WN systems; for example, the attack can authenticate malicious users for banking systems, commits identity theft, and steal web server and enterprise data [155]. Encryption algorithm, user authentication scheme, secure cookies, ciphering the packets, internet protocol security (IPSec), and SSL approaches are used to prevent session hijacking [156]. The authors of [117] discussed various techniques to avoid session hijacking, and ref. [118] provided a proxy server that overcame the session hijacking problem on the web server. Bera et al. [119] proposed a blockchain-based access control scheme that detects UAVs and mitigates session hijacking on the Internet of drone environment (IoD).
5. Application layer attacks—A user interface layer with file transfer protocol (FTP) and real-time transport protocol (RTP) delivers applications and services to the end-user to maintain QoS.
  - Simple mail transfer protocol (SMTP) attacks - Here, the connection between the mail server and the attacker’s machine is exploited, wherein the server sends the web banner (HTTP version, encoding style, server version, etc.) back to the attacker side [157] Consequently, the attackers can find valuable information about the application using this web banner. The malicious user performs this attack by sending phishing emails and spam messages. The user’s SMTP server continues sending multiple emails to other servers until that server crashes. This happens because there is no strong access control that restricts the device’s physical access, due to which the SMTP server cannot withstand the attack. Employing SSL, TLS, and inserting a security layer into the SMTP server prevents this attack [120]. To do so, Chaudhary et al. [121] proposed a cryptocurrency and blockchain-enabled email system that prevents SMTP attacks and controls mail spamming.
  - SQL injection—In structured query language (SQL) injection, attackers parse SQL commands in the SQL database and gain unauthorized access to several websites [158]. Malicious users retrieve confidential information from database tables, alter those tables, and drop it. To alleviate the risk of SQL injection, one can make more than one database user account, deploy a secure web applications firewall, limit privileges, and add input validation [159]. In [123,160], the author discussed the SQL injection prevention technique for the content delivery system, for which they used an obfuscation technique to secure their database queries. Furthermore, Tanrıverdi et al. [122] introduced a blockchain-enabled signature-based detection approach in a web attack detection application. This method detects web-based attacks and prevents SQL injection, cross-site scripting (XSS), and command injection techniques. Ref. [123] overcame the issue of SQL injection in the content delivery system.
6. Miscellaneous—Apart from the aforementioned attacks, there are some more security threats in WNs, which are as follows.
  - Impersonation attack—These attacks are performed by hacktivist and nation-state criminals that have a sophisticated agenda targeting critical organizations [126]. It can be provisioned by creating fake emails on the company site, social media, and e-commerce websites, and then the attackers can impersonate themselves by using those fake emails and accounts [161]. Moreover, they also create fake domains, unauthenticated accounts, and fraud applications to imperil the security schemes applied in the organizations. To prevent impersonation attacks, the user must avoid randomly clicking links on social media sites and emails, confirm the suspicious email request before any financial transaction, and verify a padlock icon next to a URL [162]. Ref. [163] proposed cross-technology impersonation attack prevention techniques for location-based services. Then, ref. [164] provided a solution to overcome the issue of voice impersonation attacks in smart devices.
  - Packet sniffing—This attack checks and analyze the data packets that are sent to the WNs [165]. Malicious users create data traffic and extract the packets using unencrypted mechanisms. Attackers illegally steal unencrypted data from companies, advertising agencies, and government agencies using a packet sniffing attack. Network monitoring and scanning activity, use of VPN, prevention of unsecured network, and encrypted messaging prevent packet sniffing attacks [166].
  - Evil twinning—Through a wireless access point, the attacker can configure the existing WNs [165]. The access point of the attacker’s system may have a strong signal than any other access point in the network so that the clients can choose the evil twin over the genuine access points [167]. In an evil twin attack, the malicious user creates a fake Wi-Fi network that steals the users’ personal information, log activity, and credentials. To prevent evil twin attacks, visit only secure websites, protect AP using a private security key, and take care of connecting Wi-Fi hot-spots [168].

Blockchain permits authorized users to interact with each other in a verifiable manner, and it makes digital information to be shared, viewed and stored securely [169]. In that direction, many researchers have proposed their viable security and privacy solutions for WN-based applications. For instance, in [170], Cao et al. proposed a blockchain-enabled CDMA/CA model that handles scarcity of spectrum resource issues in the wireless industrial IoT applications. They introduced stochastic mode and double-spending attack. After that, Xu et al. [171] introduced RAFT based consensus algorithm to prevent malicious jamming in IoT-based applications. Khan et al. [172] discussed 5G security for the various applications such as SDN, NFV, etc. They proposed a 5G security model for securing the network and physical layer. In [173], Bouras et al. focused on the data tempering issue in the medical field. The proposed blockchain-based identity model is for e-health scenarios.

In a similar direction, Han et al. [174] proposed blockchain-based architecture for UAVs that deal with reliability, connectivity, data privacy, and energy efficiency. Sun et al. [175] analyzed edge cache network and presented blockchain-based 6G architecture for data tampering and eavesdropping. They utilized 6G edge caching and two-hop transmission for securing the transaction. After that, Rahman et al. [176] introduced a blockchain-enabled SDN framework that reduces the end-to-end delay in resource management. Wu et al. [177] proposed blockchain-based trajectory privacy approach to protect drone communication. Moreover, in [178], the author proposed the elliptic curve Diffie–Hellman key exchange technique to protect private information in the medical field. The proposed work in [178] was further improved by Jayabalan et al. in [179] with a temper registered blockchain-based framework. They used encryption techniques, digital signatures, and hashing techniques to prevent healthcare resource loss. Fatima et al. [180] worked in the same field and prevented data tampering issues using blockchain-enabled cloud technology. Perez et al. [181] worked for crowdsensing systems and proposed smart contract-based solutions to prevent data confidentiality, integrity, and system availability issues. Then, Qahtan et al. [182] proposed the spherical fuzzy-weighted with zero inconsistency (FWZIC) method to prevent access control, user privacy, and authentication in healthcare. They introduced the grey relational analysis—a technique for the order of preference by similarity to ideal solution (GRA-TOPSIS) and the bald eagle search (BES) optimization approach for the system’s security. Table 3 presents the existing blockchain-based solution for different innovative applications, such as e-healthcare and resource allocation in security and privacy perspectives of WNs.

## 4. Blockchain: A Solution for Security and Privacy in WNs

Blockchain records information in the decentralized database (i.e., in a P2P manner) and supports immutability, becoming the critical pillar of future WN’s security and privacy. Furthermore, blockchain facilitates secure communication in sophisticated WN technologies such as virtualization, edge, open-source application programming interface (API), network slicing, cloud radio access network (RAN), etc. Toward this goal, we proposed an architecture that integrates the blockchain technology to tackle the security issues in different WN applications.

**Figure 8:**
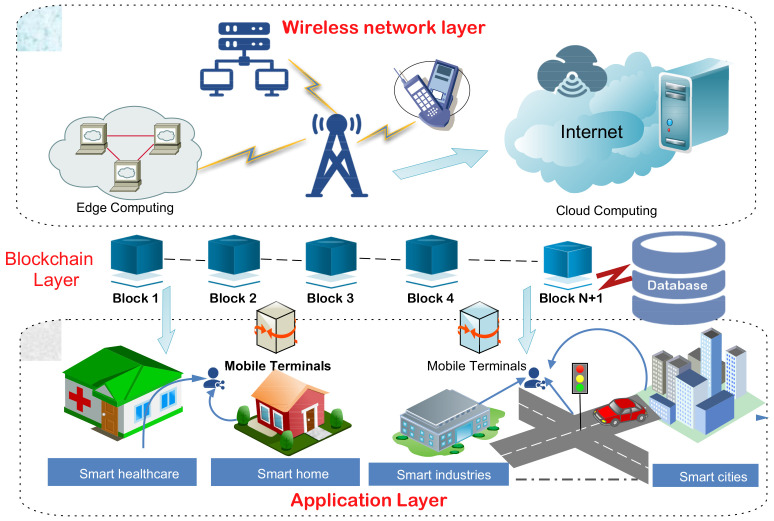
The proposed architecture: enabling blockchain technology for wireless networks.

Figure 8 illustrates the proposed architecture of WNs enabled blockchain technology. The entire architecture is divided into three different layers: (i) application layer, (ii) blockchain layer, and (iii) wireless network layer.

### 4.1. Application Layer

This layer comprises various smart applications, such as smart healthcare, smart cities, smart industries, etc. The smart application components are linked via a wired or wireless connection. In the case of wireless connection, communication happens between two users using a mobile terminal. For example, the energy bill is generated through smart meters in the smart home. The energy bill is shared with the consumer (who is consuming energy) and the smart grid administrator via WNs (e.g., 4G or 5G). During data transfer, communication is established from a user device, such as a computer or smartphone to the nearest access point of the WN layer. Then it is transferred further to the intended destination. In the proposed architecture, communication between the application and WN layers through the blockchain layer is discussed in detail in the next section.

### 4.2. Blockchain Layer

This is the middle layer in the proposed architecture, which establishes secure communication between the WNs layer and the application. First, data generated at the application layer are captured using the blockchain layer over the blocks. Once it is captured, one cannot alter it due to the immutability feature of blockchain. Then, the data are transferred from the source to the destination node securely using the WN layer. In WNs, blockchain technology offers numerous security services like access control, data integrity, and authentication, which are as follows [10]:

- Access Control: It is a physical layer security that restricts unauthorized users from accessing authorized services running on WN. The conventional access control is centralized and utilizes the standard encryption techniques that lack in providing trust in the WN application. Such a centralized system has a risk of single-point failures and privacy leakages from the key generator schemes. Therefore, as an alternative to centralized access control, trusted blockchain-based access control can help in resolving the above-mentioned issues in WN. To do that, access control permission, i.e., read and write permissions, are only granted to an authorized user, device, and machine. In addition, blockchain uses a smart contract (a set of codes to establish contracts within two parties) to secure the system against any malevolent threat [183].
- Data integrity: Data integrity is another such issue where the attackers tamper with the data of the smart application. As a consequence, the falsified data can mislead the behavior of the smart application. Therefore, storing the data inside the blockchain can ensure that the data are not manipulated. Furthermore, it performs data integrity verification of both the communicating parties by auditing all the transactions that occurred between them [184].
- Authentication: blockchain incorporates authentication capabilities to increase the robustness of the network, which detects and prevents malicious activity in the network resources. Smart contracts perform request authentication to avoid unauthorized access from malicious users [184]. Moreover, it offers a secure and authenticated environment to create virtual WNs (VWNs). Using this network, wireless resource owners can rent their resources, such as infrastructure and a slice of the RF spectrum, to the mobile virtual network operator [10].

### 4.3. Wireless Network Layer

This layer comprises various 6G services across several vertical sectors, such as vehicle-to-vehicle (V2V), D2D, virtual reality (VR), augmented reality (AR), video streaming, and collaborative gaming to the users residing in the application layer. In addition, it also consists of breakthrough technologies, for instance, SDN, NFV, cloud computing, and many more, that assist in meeting the significant specification of future WNs. The aforementioned services use the precarious wireless networks that hinder the performance of the 6G-based WN applications. This layer plays an important role in establishing a secure connection between sender and receiver using the blockchain layer. Enabling blockchain in WNs can ensure security and reliability in the network by securely storing the data in a distributed manner, i.e., no single stakeholder controls the data; the data are distributed to all the authenticated members of the blockchain. Then, the stakeholder requires a smart contract to establish a service level agreement (SLA) with communicating parties to place the 6G services on lease or share it. The smart contract also automates the resource allocation process (resources such as channel, spectrum, and power) and network orchestration that involve several stakeholders across the entire WNs to provide smooth and transparent service to end-users. Blockchain as the whole process is secure, reliable, and auditable. This integration of blockchain and WNs deliver services that create several other challenges, such as network resiliency, robustness, and data integrity.

## 5. Result Analysis

This section elaborates on the experimentation details and result analysis of the proposed architecture in terms of scalability, latency, and packet-loss ratio.

### 5.1. Experimental Setup And Tools

The blockchain and WN are simulated in two different platforms to accomplish a shared objective, i.e., to provide essential 6G services to the application layer. For blockchain, we used the Remix integrated development environment (IDE) and solidity programming language to create digital smart contracts, which helps eradicate the intermediate third-party services and improves the operation of the blockchain network. Furthermore, to provide the benefits of a 6G network, we mimicked the MATLAB-based 5G toolbox by modifying its parameters to behave as a 6G network. The parameters, such as frequency range (95 GHz), channel bandwidth (130 MHz), subcarrier spacing (60 kHz), and many more, are changed in the 5G toolbox. A particular user of the application layer demands the 6G-based service from the WN layer; once the user accesses the 6G services, they can communicate with each other for a particular task (smart home/smart cities/smart healthcare, etc.) of the application layer. Moreover, the blockchain secures the data communication between the application and the WN layer by securely storing the data in the immutable ledger. The proposed architecture is evaluated against different performance metrics, such as scalability, packet loss ratio, and latency. The entire architecture is simulated in a desktop that has configuration, such as Intel core i5, 16 GB RAM, 250 GB SSD, and Intel iRIS graphic card. Due to the involvement of high-end computing software, i.e., MATLAB 2021, and RemiX IDE this configuration is a minimal configuration needed to simulate the proposed architecture. Additionally, Table 4 shows the different simulation parameters that are used in the proposed architecture.

### 5.2. Performance Evaluation

**Figure 9:**
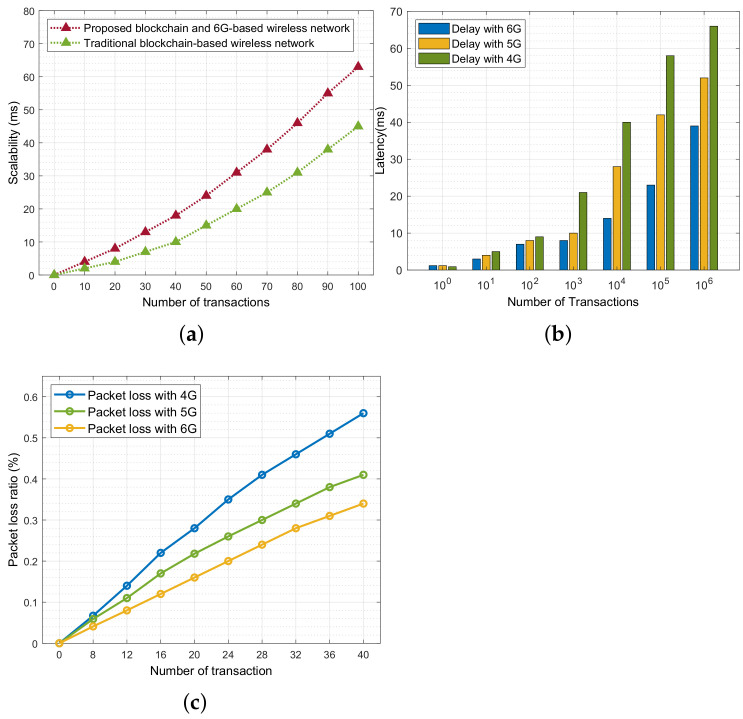
Result analysis of the proposed architecture. (**a**) Scalability comparison. (**b**) Latency comparison. (**c**) Packet-loss ratio comparison.

Figure 9a shows the scalability comparison between the traditional WN and blockchain and 6G-based WN. The traditional WN (4G and 5G) offers high data rates (10 Gbps), low latency (10 ms), and better availability (99.999%); despite this, it is insecure against various security threats that degrade the performance of WN. Contrary, the indispensable characteristic of a 6G network brings improvement in the properties of WN, such as ubiquitous data rates (1 Tbps), low latency (1 ms), high mobility support (1000 km/h), high availability (99.99999%), and high scalability ($10^{9}$ devices/sqm). Due to such competency in a 6G network, it is incorporated into the proposed architecture. As a result, the proposed blockchain and a 6G-based WN architecture offer higher scalability than the conventional WN. Furthermore, the 6G network improves network latency and data rates, resulting in a minimal per unit time to store the transaction block compared to the conventional WN. Therefore, it raises the transactions in the blockchain network and augments the network scalability. From the graph, it is evident that the traditional WN has lower scalability due to lower latency and data rates and high per unit time of transaction block compared to a blockchain and a 6G-based WN.

Figure 9b displays the latency comparison among different conventional WN (4G and 5G) and a 6G-based WN. It is clear from the graph that the 6G-based WN has better latency as compared to the traditional generation of WN, i.e., 4G and 5G. This is because the incorporation of a 6G network propels the WN to offer peak data rates (1 ≥ Tbps), higher mobility (≥1000 km/h), low latency (10–100 μs), high end-user data rate (1 Gb/s), dense connectivity ($10^{7}$ devices/km${}^{2}$), and higher availability (99.99999%). Consequently, it not only improves the overall performance of WN but also enhances the end-user experience of the application layer in the proposed architecture. The integration of the 6G network not only improves the overall performance of the WN but also improves the scalability of the proposed architecture. Figure 9c illustrates the comparison of packet loss ratio among different conventional WN (4G and 5G) and the proposed blockchain and a 6G-based WN. As discussed, with the essential benefits of a 6G network in the aforementioned section, it is evident that there is a minimal data packet loss in the communication between the application and WN layer of the proposed architecture. From the graph, we can infer that the 6G-based WN has a lower packet loss ratio than a 4G and 5G network. This also motivates to maximize the scalability of the proposed architecture because only a few packets are dropping from the communication, and most of the data packets are securely stored inside the blockchain ledger.

## 6. Open Issues and Research Challenges

**Figure 10:**
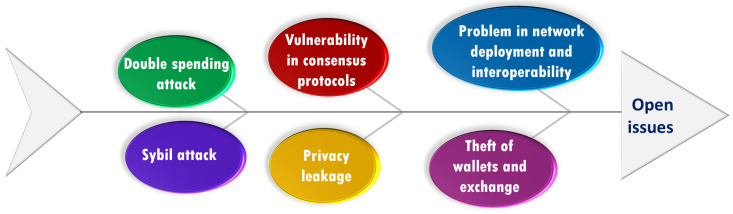
Open issues and research challenges.

Blockchain is one of the promising technology to make a revolution in WNs. It has the advantages of transparency, traceability, decentralization, and high-security feature. However, there are some open research challenges and issues available while integrating the blockchain with WN. Figure 10 presents open security and privacy issues and research challenges in blockchain-enabled future WNs. Security mechanisms provided by 3GPP have trustworthy links for non-malicious radio connectivity, but they cannot defend against DDoS and radio-electronic-based attacks. Blockchain nodes, consensus mechanisms, smart contracts, and wallets are key elements for enabling security in WN; however, attackers can find the core vulnerabilities from these elements and manipulate them. A summarized explanation of such open issues of blockchain-based WNs is discussed in detail as follows.

- Double spending attack comprises spending of a single token multiple times by user over blockchain, which needs to be handled to secure WNs [3].
- Transactions are calculated in blockchain before they are accepted or rejected, which is done using consensus protocols. It is vulnerable to malicious activity, so it requires to be tested before deploying in the large-scale WN environment [14].
- Unauthenticated users can access wallets and exchanges using DDoS attacks. They successfully perform the theft of wallets.
- Malicious user controls more than 50% of nodes known as 51% attacks in the blockchain. Therefore, an attacker would be able to manipulate a user’s information by introducing this type of attack [10].
- Sybil attack runs multiple malicious nodes on the network. They make restrictions for new nodes to access and add new blocks in the network [185].
- The problem of network deployment and interoperability comes when blockchain deploys in 5G and beyond networks with SDN, NFV, and MEC technologies that need to mitigate future WNs.
- Blockchain and smart contract in WNs facing leakage of transaction data privacy, smart contract logic privacy, and user privacy, which is also grabbing the attention of researchers [186].
- In permissioned blockchain, when nodes join the network, the system needs to ensure authenticity and authority, which raises concern for authentication and control over data [14].

Some more research issues related to future WNs are also discussed that give future directions for further investigations, which are as follows.

- In LTE networks, payload and control signalling are required to be encrypted, and the ratio between the amount of data transmitted and encryption overhead needs to be considered [187].
- Memory and resource consumption in large-scale networks is enormous. In addition, data collection, filtering, data sampling, and pre-processing are challenging because of heterogeneous data, which require security assurance and privacy protection [188].
- MIMO fulfills the requirements of services and data rate, but from a security point of view, resource allocation in MIMO needs to be elaborate.
- To improve network traffic and security, it requires further investigations in beamforming [43].

## 7. Conclusions

In the early stage of WNs evolution, many studies have proposed providing security and privacy solutions for the WNs. However, with the evolution of WNs, the security implications are highly maneuvered to exploit different WN-based intelligent applications, such as smart homes, smart cities, intelligent transportation systems, and many more. In that viewpoint, blockchain has revolutionized numerous applications by moving beyond cryptocurrency. As future WNs are expected to be more secure and resilient, blockchain-based WNs are becoming a booming research domain to address such security issues as authentication, data manipulation, tractability, and network performance. This paper provides a decade survey of past and current research work that has been carried out for the security and privacy issues in WNs. We also propose a taxonomy exploring the security and privacy issues at each layer of the OSI model with their countermeasures based on the detailed analysis. Further, we highlight the competency and necessity of blockchain technology by creating a blockchain and a 6G-based WN architecture to confront the security threats of WN. Then, the proposed architecture is assessed by different evaluation metrics, such as packet loss ratio, scalability, and latency. Finally, the paper concluded with a discussion on open issues and research challenges in blockchain-based WNs.

In future work, based on the proposed survey, we will build a novel solution to tackle modern security threats, such as Sybil attacks, malware, and double spending, to strengthen the security and privacy of WNs.

## Figures and Tables

**Table 1:** A comparative analysis of the existing survey with the proposed study on the blockchain-based solutions for the security and privacy issues in WNs.

Year	Author	Contribution	Observations	Remarks
2016	Zou et al. [32]	Discussed security attacks, protocols, and algorithms in WNs for physical layer	Discussion about security and privacy in other layers have lacked	Cover security and privacy in every layer
2017	Boudguiga et al. [58]	Discussed blockchain infrastructure for IoT	Analyzed confidentiality, integrity and availability with different use case	Did not discuss the vulnerability in the security
2018	Ferrag et al. [59]	Analyzed existing privacy-preserving and authentication schemes for 4G and 5G	Did not discuss layer-wise security in WNs	Study on layer-wise security for different generation networks is required
2019	Pohrmen et al. [60]	Explored IoT-based system with Blockchain for SDN platforms	Application of blockchain in subsystem is lacking	Blockchain usage in smart applications, such as smart city, autonomous vehicle, identity management, etc., that need emphasis
2019	Casino et al. [61]	Review of blockchain-based applications through multiple domains, established trends, key themes, and emerging areas	Security requirement in different application domain has not been covered	Analyzed security requirements using blockchain with scalability, privacy, interoperability, audit, latency, and visibility issues
2019	Rouhani et al. [62]	Reviewed existing approaches and security tools for performance enhancement of decentralized applications based on smart contract	Did not discuss problems and limitations of smart contract in individual applications	A comparison of blockchain-based solutions with other existing solutions is required
2019	Wang et al. [65]	A Blockchain scheme for IoT	Comparison of existing security techniques with blockchain techniques is missing	Should provide a comparative analysis of existing techniques with blockchain-based approaches
2019	Aggarwal et al. [63]	Surveyed Blockchain for smart communities and analyzed process models for secure transactions	Comparison of Blockchain with existing security solutions is lacked	Survey on blockchain-based security and privacy challenges needs to be highlighted
2019	Hussien et al. [74]	Studied blockchain in healthcare domain with various features such as data integrity, access control, etc.	Comparison of consensus mechanism of blockchain is not included	Should emphasize application of blockchain for security and privacy perspective
2020	Liu et al. [64]	A blockchain-based identity management solutions	Highlighted conceptual analysis for identity management solutions	Real-time applicability of blockchain-based solution need to be taken into consideration
2020	Xie et al. [66]	Discussed blockchain for cloud data exchange based on transaction management, and reputation systems	Analysis of existing security methods with blockchain for cloud is lacking	Existing blockchain-based solutions for cloud data exchange need to be discussed in detail
2020	Tahir et al. [14]	Discussed blockchain in 5G and future WNs	Did not cover comparison of blockchain with another approaches of security and privacy	Cover existing security and privacy solutions based on blockchain
2021	Wazid et al. [67]	Examined the existing research in the field of 5G-enabled IoT communication environment and provided system model, security protocol, and attacks	Did not cover the detail about layer-wise security in WNs	Layer-wise security in WNs should be considered
2021	Hewa et al. [68]	Explored the applications for the blockchain-based smart contracts	Application of smart contract in security and privacy perspective is lacking	Should cover existing method and compare with smart contract-based with application
2022	Saraswat et al. [69]	Presented blockchain enabled federated learning in UAVs for trusted exchange of information	Discussed UAV security with blockchain and proposed case study	Security attacks for blockchain enabled UAV need to be highlighted
2022	Abualsauod et al. [70]	Studied blockchain and ML techniques for the security in UAVs	Analyzed the hybrid blockchain model for security and reliability and presented future direction	Real-time applicability needs to be discussed with case study
2022	Grover et al. [71]	Studied security of ITS in VANET	Discussed existing solution based on blockchain for VANET security	Did not consider layer-wise security attacks
2022	Pattewar et al. [72]	Analyzed existing solution for IoT security using blockchain	Discussed consensus algorithms for security and privacy in various use case	Did not specify the security vulnerability and attacks
2022	Wang et al. [73]	Studied security and privacy issues in metaverse	Analyzed security threat and blockchain based security solutions	Did not consider possibility of the security attacks
2022	The proposed approach	Analyzed security requirements and issues based on different layer, proposed taxonomy for existing security and privacy approaches envisioned security attacks and their countermeasures	Proposed a blockchain-based WNs architecture for security and privacy perspective with the scalability and latency comparison	-

**Table 3:** Blockchain-based solution for different smart applications in security and privacy perspectives of WNs.

Year	Author	Applications	Security Issues	Approach	Pros	Cons
2020	Cao et al. [170]	Wireless industrial IoT (IIoTs)	Scarcity of spectrum resources	Presented blockchain-based on CDMA/CA model	Introduced stochastic mode and double-spending attack on CDMA/CA	Not considered layer wise security
2020	Xu et al. [171]	IoT applications	Malicious jamming	Presented RAFT based blockchain network	Proposed consensus algorithm	Not considered multi-jammer problem
2020	Khan et al. [172]	5G security for different technology like SDN, NFV etc	Detection and prevention of sabotage	Proposed 5G security model	Considered network and physical layer security	Other layer security is lacked
2020	Bouras et al. [173]	e-healthcare	Data tampering	Analyzed eHealth scenario using blockchain	Presented identity model with blockchain	Did not consider real-time scenario results
2021	Han et al. [174]	Unmanned aerial vehicles	Reliability and connectivity, energy efficiency, data privacy	Blockchain-based approach	Proposed drone-based architecture on 5G for with different technologies	Did not discuss security attacks
2021	Sun et al. [175]	Edge cache network	Data tempering and eavesdropper	Blockchain-based 6G framework	Used 6G edge caching and two-hop transmission, optimize the cashing for secure transaction	Did not incorporate a comparative analysis of the proposed approach with existing security solutions
2021	Rahman et al. [176]	Resource management for IoT	Energy efficiency and end-to-end delay for resource utilization	Blockchain-based SDN framework	Proposed cluster-head selection algorithm to secure network	Did not consider mobility
2021	Wu et al. [177]	Drone Communication	Drone ID management, privacy and trajectory protection	Presented blockchain-enabled trajectory privacy protection scheme	Used in military and civilian area	Did not cover security attacks
2021	Wu et al. [178]	Healthcare	Private information protection of medical system	Elliptic curve Diffie–Hellman key exchange approach and file authorization contracts	Enhanced reliability and bandwidth utilization of data transmission	Did not discuss security attacks on the existing approach
2022	Jayabalan et al. [179]	Healthcare	Monetary and resource loss	Temper register blockchain bad framework with IPFS storage for the healthcare	They used symmetric and asymmetric key encryption, digital signature and hashing algorithm and IPFS to deal with scalability issue	Did not discuss security and privacy attacks in the system
2022	Fatima et al. [180]	Healthcare	Data tampering	Blockchain-based cloud technology for healthcare	They presented state of the art on blockchain enabled healthcare system	Did not consider security issues of the existing solution
2022	Perez et al. [181]	Crowd-sensing systems	Data confidentiality, integrity, and system availability	Proposed crowd-sensing systems using smart contracts and blockchain	They discussed security and privacy issue with blockchain based solution in crowd-sensing systems	Did not discuss security and privacy problem to add blockchain and smart contract in the system
2022	Qahtan et al. [182]	IoT healthcare Industry 4.0 systems	User authentication, access control, and privacy protection	Analyzed MCDM problem in blockchain enabled IoT healthcare Industry 4.0 systems	Proposed spherical FWZIC for security and privacy, GRA-TOPSIS, and BES approach for optimization	Did not discuss security and privacy attacks and their countermeasures in the presented approach

**Table 4:** Simulation parameters of the proposed architecture.

Parameters	Values
Wireless network layer parameters	
Frequency range	95 GHz
Channel bandwidth	130 MHz
Subcarrier spacing	60 KHz
Modultation	OFDM
Channel coding	Polar coding
Fading channel	comm.RayleighChannel
Blockchain layer parameters	
Solidity compiler	0.8.10+commit.fc410830
Remix environment	Injected Web3
Gas limit	3,000,000

## Data Availability

Not applicable.

## Abbreviations

The following abbreviations are used in this manuscript:

1G	First Generation
2G	Second Generation
3G	Third Generation
3GPP	Third Generation Partnership Project
4G	Fourth Generation
5G	Fifth Generation
AMF	Access and Mobility management Function
ARQ	Automatic Repeat Request
CDMA	Code-Division Multiple Access
CRC	Cyclic Redundancy Check
DoS	Denial of Service
D2D	Device to Device
eNodeB/eNB	Evolved Node B
EPC	Evolved Packet Core
EPS	Evolved Packet System
ERLLC/eURLLC	Ultra-Reliable Low Latency Communications
E-UTRAN	Evolved UMTS Terrestrial Radio Access Network
FeMBB	Further Enhanced Mobile Broadband
FEC	Forward Error Correction
FL	Federated Learning
GSM	Global System for Mobile Communications
GUTI	Global Unique Temporary Identifier
HARQ	Hybrid Automatic Repeat Request
IDM	Identity Management
IMS	IP Multimedia Subsystem
IMSI	International Mobile Subscriber Identity
IMT	International Mobile Telecommunications
IoE	Internet of Everything
IoMT	Internet of Military Things
ITU	International Telecommunications Union
LTE	Long-Term Evolution
MAC	Medium Access Control
MIMO	Multiple Input Multiple Output
MTC	Machine-Type Communications
NAS	Non-Access Stratum
NFV	Network Function Virtualization
OFMD	Orthogonal Frequency Division Multiplexing
P2P	Peer-to-Peer
QoS	Quality of Service
RAN	Radio Access Network
RFID	Radio Frequency Identification
RTP	Real-Time Transport Protocol
SC	Smart Contract
SDN	Software defined Network
STBC	Space Time Block Coding
SUPI	Subscription Permanent Identifier
SUPI	Subscription Permanent Identifier
TCP	Transmission Control Protocol
UDP	User Datagram Protocol
umMTC	Ultra Massive Machine-Type Communications
WEP	Wired Equivalent Privacy
WiMAX	Worldwide Interoperability for Microwave Access
WN	Wireless Network
WPA	Wi-Fi Protected Access
